# Efficacy of Total En Bloc Spondylectomy versus Stereotactic Ablative Radiotherapy for Single Spinal Metastasis

**DOI:** 10.3390/cancers15235518

**Published:** 2023-11-22

**Authors:** Dong-Ho Kang, Wooseok Lee, Bong-Soon Chang, Hyoungmin Kim, Sam Yeol Chang, Seong Hwa Hong, Jin Ho Kim, Hee Jung Son

**Affiliations:** 1 Department of Orthopedic Surgery, Seoul National University College of Medicine, Seoul National University Hospital, 101 Daehangno, Jongno-gu, Seoul 03080, Republic of Korea; kang9451@gmail.com (D.-H.K.); dldntjr1594@gmail.com (W.L.); honginsnuh@gmail.com (S.H.H.); 2 Department of Radiation Oncology, Seoul National University College of Medicine, Seoul National University Hospital, 101 Daehangno, Jongno-gu, Seoul 03080, Republic of Korea; 3 Department of Orthopedic Surgery, Nowon Ulji University Hospital, 68 Hangeulbiseok-ro, Nowon-gu, Seoul 03080, Republic of Korea; sonthespine@gmail.com

**Keywords:** total en bloc spondylectomy, stereotactic ablative radiotherapy, spinal metastasis

## Abstract

**Simple Summary:**

Spinal metastases denote the spreading of cancer into the spinal canal and causing of spinal cord compression. We compared the complete surgical removal of one or more vertebrae above the sacrum (total en bloc spondylectomy; TES) with the use of high radiation dose (stereotactic ablative radiotherapy; SABR) to eliminate spinal metastases. A total of 38 matched patients were analyzed (19 TES, 19 SABR) and the median follow-up period was 54.4 months (TES) and 26.1 months (SABR). Two-year progression-free survival (PFS) and overall survival (OS) rates were 66.7% and 78.9% in the TES group and 38.9% and 50.7% in the SABR group, respectively. The two matched groups showed no significant differences in OS and PFS. The rate of major complications was higher in the TES group than in the SABR group (21.1% vs. 10.5%). SABR resulted in fewer complications compared to TES, whereas TES demonstrated superior mid-term metastatic tumor control.

**Abstract:**

To compare total en bloc spondylectomy (TES) with stereotactic ablative radiotherapy (SABR) for single spinal metastasis, we undertook a single center retrospective study. We identified patients who had undergone TES or SABR for a single spinal metastasis between 2000 and 2019. Medical records and images were reviewed for patient and tumor characteristics, and oncologic outcomes. Patients who received TES were matched to those who received SABR to compare local control and survival. A total of 89 patients were identified, of whom 20 and 69 received TES and SABR, respectively. A total of 38 matched patients were analyzed (19 TES and 19 SABR). The median follow-up period was 54.4 (TES) and 26.1 months (SABR) for matched patients. Two-year progression-free survival (PFS) and overall survival (OS) rates were 66.7% and 72.2% in the TES and 38.9% and 50.7% in the SABR group, respectively. At the final follow-up of the matched cohorts, no significant differences were noted in OS (*p* = 0.554), PFS (*p* = 0.345) or local progression (*p* = 0.133). The rate of major complications was higher in the TES than in the SABR group (21.1% vs. 10.5%, *p* = 0.660). These findings suggest that SABR leads to fewer complications compared to TES, while TES exhibits better mid-term control of metastatic tumors.

## 1. Introduction

Spinal metastases are the most common metastatic skeletal diseases, accounting for approximately 60% of all cases [1,2]. The overall cumulative incidence of clinically diagnosed spinal metastasis in patients with solid tumors is 15.67%, and about 10% of patients with spinal metastasis eventually develop metastatic epidural spinal cord compression (MESCC) [3]. After the publication of the Patchell study, the standard care for MESCC has evolved to include decompressive surgery followed by conventional radiotherapy [4]. Surgical or radiological treatments that allow for local tumor control and survival have played a critical role in the clinical management of spinal metastases with or without MESCC and have improved the life expectancy of cancer patients [5,6,7]. Stereotactic ablative radiotherapy (SABR) delivers a high radiation dose to the targeted area while minimizing exposure to adjacent normal tissues, including the dural sac [8,9,10,11,12,13,14,15]. Owing to its effectiveness in ablating radioresistant tumors, SABR has brought about a paradigm shift in cancer treatment for spinal metastases [15,16].

The oligometastatic state, an intermediate phase between manageable localized disease and inoperable extensive disease, has shown excellent control rates when spinal involvement is treated with SABR [17,18]. This is supported by the findings of recent randomized trials indicating overall survival and progression-free survival benefits compared with conventional radiation therapy [19]. In contrast, traditional total en bloc spondylectomy (TES) for spinal metastasis has shown mixed oncological outcomes in terms of curability [6,15,20,21], with potential disadvantages such as surgical site infection and pneumonia [22]. A current review has reported an average postoperative survival of 15.2 months for patients with isolated spinal metastasis after TES, underlining the need to explore further optimal treatment approaches [23].

While previous studies on TES and SABR have reported on efficacy in achieving good local control rates and improving survival rates, information comparing the efficacy of the two different types of treatments is still lacking. No studies have directly compared TES and SABR for treating spinal metastases and none have been conducted for other types of cancer [24]. The purpose of this study was to compare SABR and TES in patients with single spinal metastasis by assessing local progression, overall survival, and postoperative complications.

## 2. Materials and Methods

### 2.1. Study Design

This retrospective study aimed to compare the treatment outcomes of TES and SABR for single spinal metastasis. This study was approved by our institutional review board. Patients with single spinal metastasis who underwent TES between 2002 and 2015 or SABR between 2012 and 2019 were included in the study. Single spinal metastasis was defined as a lesion of spinal metastasis confined to one vertebral body, regardless of other metastases to internal organs or extraspinal bone. Patients with multiple spinal metastases, previous spinal tumor resections, or sacral metastasis were excluded. The SABR program for spinal metastases commenced at our institution in 2012. Medical records, as well as magnetic resonance (MR) and computed tomography (CT) findings, were retrospectively reviewed. Data on patients and cancer characteristics, New England Spinal Metastasis Score (NESMS), modified Tokuhashi score, Tomita’s surgical classification, Spinal instability neoplastic score (SINS), treatment details including adjuvant chemotherapy, and details of SABR and TES were collected. To compensate for the heterogeneity between those who received SABR and those who received TES, patients in the TES group were matched with those who received SABR. Treatment outcomes were compared among matched patients. For between-group comparisons, we only used follow-up data collected up to 97.7 months in the TES group, which was the longest follow-up period in the SABR group. An institutional statistical consultation recommended that the small number of patients and events precluded the use of propensity score matching for the current study. Accordingly, patients from the SABR and TES groups were matched in a 1:1 exact ratio with similar primary tumor sites. Each patient who underwent TES was matched with one patient who underwent SABR for the same primary tumor type. If the corresponding identical origin of the tumor was unavailable between the groups, matching was performed between the different tumor types with similar median survival periods, as reported previously [25]. After the tumor origin was matched, the patients were further matched using identical NESMS and modified Tokuhashi score ranges.

### 2.2. Surgery and Radiotherapy

**Figure 1:**
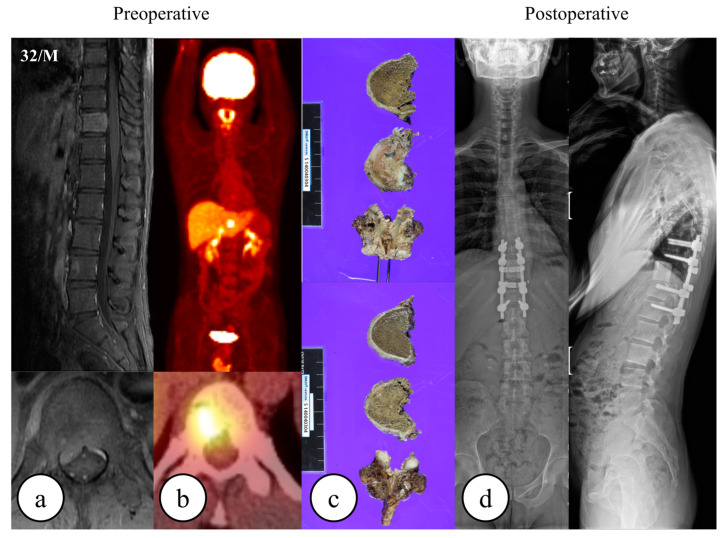
A 32-year-old male underwent total en bloc spondylectomy for a solitary metastasis of adrenal cortical carcinoma at T11. The following images are provided: (**a**) preoperative MRI, (**b**) preoperative whole-body FDG-PET CT, (**c**) gross pathology, and (**d**) postoperative radiography.

**Figure 2:**
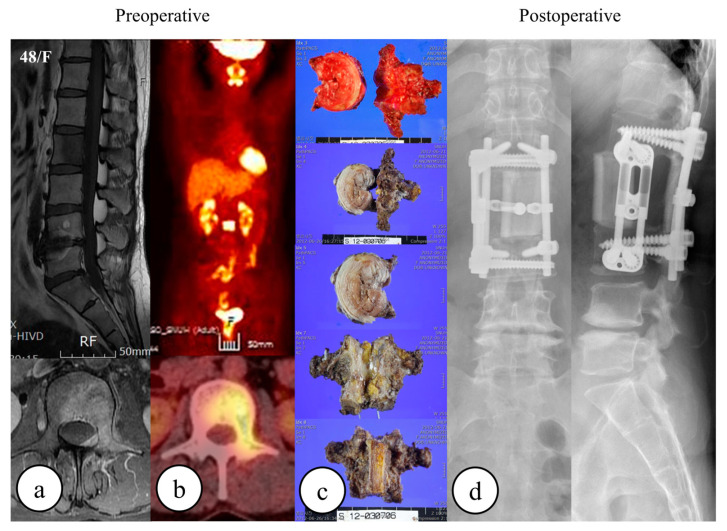
A 48-year-old female underwent total en bloc spondylectomy for a solitary metastasis of breast cancer at L2. The following images are provided: (**a**) preoperative MRI, (**b**) preoperative whole-body FDG-PET CT, (**c**) gross pathology, and (**d**) postoperative radiography.

All patients treated via TES underwent preoperative embolization of the segmental arteries of the tumor-affected vertebra. Patients underwent endotracheal general anesthesia and were positioned prone for the posterior approach. Following sterilization, a midline dorsal incision was made, the lumbar fascia was divided, and the paraspinal muscles subperiosteally were dissected from the spinal and transverse processes, with subsequent lateral retraction of the paraspinal soft tissues. Pedicle screws were inserted bilaterally, guided by intraoperative radiography. A transpedicular osteotomy was performed utilizing a fine-thread saw. In thoracic cases (Figure 1), rib resection (~2 cm) facilitated thoracic cavity access and segmental nerve root ligation. For lumbar cases (Figure 2), in certain instances, a retroperitoneal approach was adopted following posterior surgery, while in others, the procedure was confined to the posterior aspect alone. A plane was created lateral to the vertebral body and medial to the psoas to safeguard traversing nerve roots. Posterior decompression involved the removal of bilateral pedicles, laminae, transverse processes, and spinous processes. A plane was bluntly dissected between the vertebral body and major vessels, followed by adjacent-level discectomies. The affected vertebra was removed, followed by the placement of either an allograft strut or a titanium mesh, filled with autogenous or allograft bone, into the intervertebral space. Posterior instrumentation, optionally complemented by anterior instrumentation, was employed to ensure the stability of the graft or mesh. Intralesional resection is defined as the incidental or deliberate breach of the tumor by the surgeon during the procedure. Patients treated via TES underwent postoperative radiotherapy unless it was contraindicated. Postoperative radiotherapy was administered after a mean value of 4 weeks post-surgery. Patients in the SABR group received exclusive SABR treatment, devoid of any surgical interventions, including separation surgery or laminectomy. The SABR protocol at our institution has been described previously [26]. Briefly, CT and MR planning images were obtained for use in conducting the SABR treatment. All clinical target volumes were delineated by a radiation oncologist (JHK) according to the recommendations of the International Spine Radiosurgery consortium [27]. Any epidural extension of the tumor was explicitly included in the target volumes. Organs at risk were delineated on CT images superimposed on to MR images. A 1–2 mm margin was added to the target volumes and organs at risk to compensate for random and systematic errors. SABR was delivered using onboard image guidance. Patients were followed-up regularly with MR images after SABR was delivered.

### 2.3. Endpoints

Local control of the treated spinal segments, progression-free survival, and overall survival were compared between the matched TES and SABR groups. These endpoints were calculated from the date of TES or completion of SABR.

Local progression was defined as tumor regrowth within the treated spinal segments. The diagnosis of local progression of spinal metastatic lesions entailed unequivocal findings in at least one imaging study such as radiography, MR, CT, bone scan, and positron emission tomography. Progression was defined as death or unequivocal tumor progression at any anatomical site. Overall survival was determined using death notification data from the Ministry of Public Administration and Security. Ambulatory function was evaluated using survival analysis to compare the proportion of patients maintaining an Eastern Cooperative Oncology Group (ECOG) performance status of 2 or lower between the TES and SABR groups.

Major complications were classified as Grade 3 or higher according to the common terminology criteria for adverse events [28].

### 2.4. Statistical Considerations

Differences in continuous and categorical data were evaluated using Student’s *t*-test or Mann–Whitney test, and the Chi-squared or Fisher’s exact test. Survival estimates were obtained using the Kaplan–Meier method and compared using the log-rank test. Factors associated with local progression, such as age, follow-up period, American Society of Anesthesiologists (ASA) score, ECOG scale, primary cancer group of modified Tokuhashi score, number of extraspinal bone metastasis, status of internal organ metastasis, radioresistance of primary tumor, history of prior radiotherapy, location of spinal metastasis, history of prior adjuvant treatment, radiation scheme, Bilsky grade of spinal metastatic lesion, size of the lesion, and time from diagnosis to treatment were examined using a Cox regression model. IBM SPSS statistics software version 25.0 (IBM Corp., Armonk, NY, USA) was used for the statistical analysis.

## 3. Results

A total of 89 patients were included in our study, with 20 undergoing TES and 69 undergoing SABR. TES was implemented with the goal of wide excision and radical treatment, but intralesional resection was performed in 5 patients (25%). Among the 20 patients who underwent TES for single spinal metastasis, one patient was excluded from matching because of the existence of a double primary tumor. Ultimately, 19 patients in the TES group were matched with 19 patients in the SABR group. The demographic and oncological characteristics of all matched patients are shown in Table 1 and File S1. In cases where the tumor origins could not be matched exactly as per the aforementioned criteria, poorly differentiated carcinoma and adrenocortical carcinoma were matched with hepatocellular carcinoma (HCC), and breast cancer was matched with thyroid cancer. In the matched cohorts, the SABR group’s patients were significantly older than those in the TES group. The mean follow-up period was 36.3 months (SD, 30.9 months), and the TES group had a significantly longer follow-up period than the SABR group for all patients; however, the difference was not significant in matched patients. The proportion of male patients in the SABR group was significantly lower than that in the TES group. The prevalence of patients with an ASA score of 1 was significantly higher in the SABR group compared to the TES group in all patients. Other demographic and oncological data showed no significant differences between the two groups in either all patients or matched patients alone. Detailed information regarding the radiation schemes for patients in both the TES and SABR groups is presented in Table 2.

**Figure 3:**
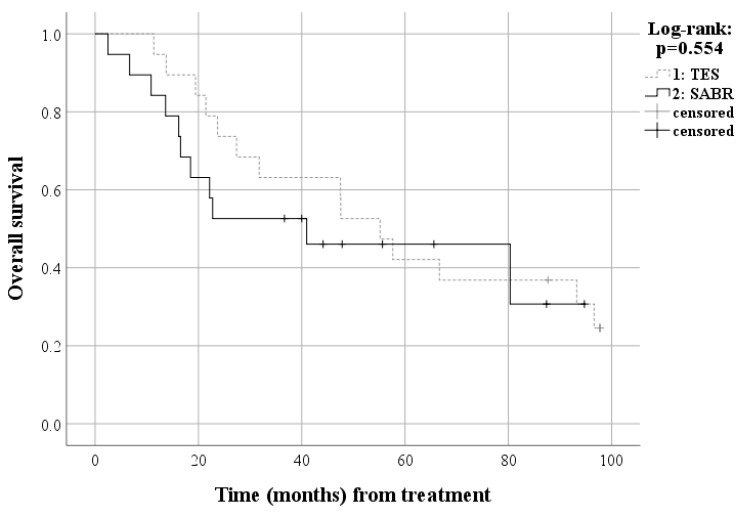
Survival curve for overall survival among matched patients. TES, total en-bloc spondylectomy; SABR, stereotactic ablative radiotherapy.

**Figure 4:**
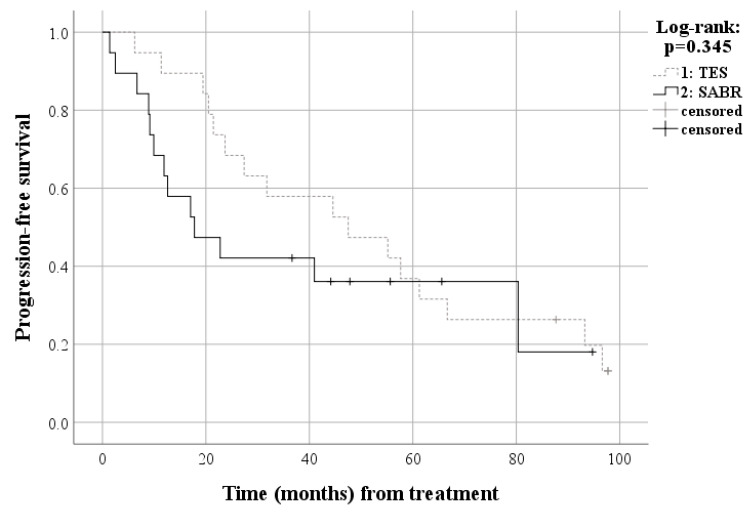
Survival curve for progression-free survival among matched patients.

**Figure 5:**
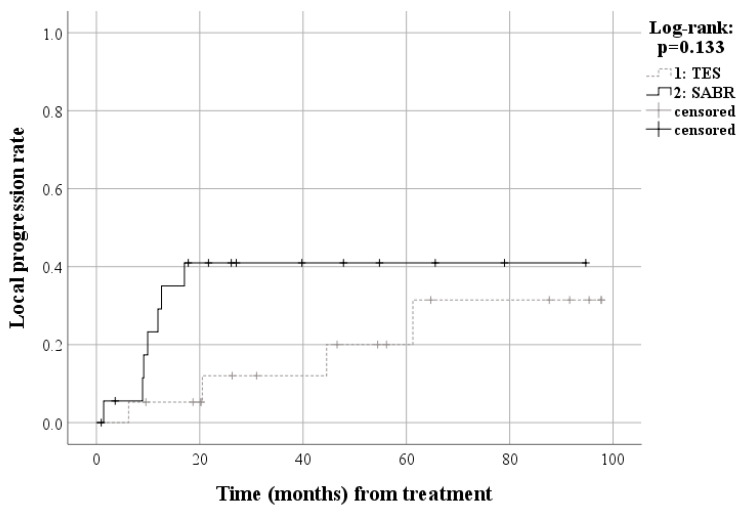
Survival curve for local metastasis progression among matched patients.

**Figure 6:**
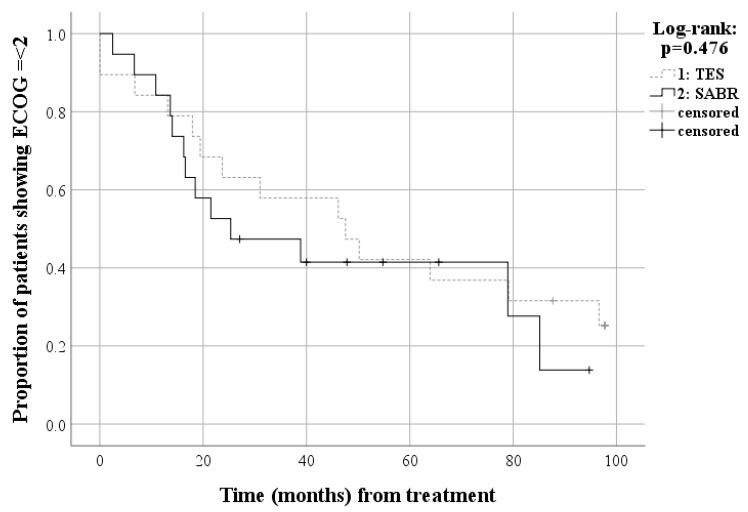
Kaplan–Meier curve depicting the proportion of matched patients maintaining an ECOG performance status of 2 or lower.

In matched patients, 14 patients (73.7%) died due to disease progression and five patients (26.3%) are alive after TES with an estimated median survival of 55.2 months (95% Confidence Interval [CI], 40.8–69.6 months), including three patients (15.8%) with no evidence of disease and two patients (10.5%) with disease. Meanwhile, 11 patients (57.9%) died due to disease progression and eight patients (42.1%) were alive after SABR with an estimated median survival of 41.0 months (95% CI, 0.0–90.3 months), including two patients (10.5%) with no evidence of disease and six patients (31.6%) with disease. The cumulative 24-month overall survival rate was 78.9% in the TES and 50.7% in the SABR group. Survival analysis of the matched patients also showed no significant difference for overall survival between the TES and SABR groups (*p* = 0.554) (Figure 3). The two-year local progression-free survival rates were 66.7% in the TES group and 38.9% in the SABR group. Survival analysis of the matched patients also showed no significant difference in progression-free survival between the TES and SABR groups (*p* = 0.345) (Figure 4). The cumulative 24-month local progression rate among the matched patients was 12.8% in the TES and 43.5% in the SABR group. Survival analysis, confined to a two-year follow-up, revealed a statistically significant difference in local progression rates between the TES and SABR groups (*p* = 0.04). However, when analyzing matched patients up to the final follow-up, there was no significant difference in local progression between TES and SABR groups (*p* = 0.133) (Figure 5). The Kaplan–Meier curve comparing the proportion of patients with an ECOG performance status of 2 or lower revealed no significant difference between the TES and SABR groups (*p* = 0.476) (Figure 6). Two patients in the TES group were treated with conventional chemotherapy instead of the more recent targeted therapies (File S3).

There was no significant difference in the major complication rates between the TES and SABR groups (*p* = 0.660). In the case of TES, four patients (21.1%) developed major complications after TES including distal ureter injury in one patient, postoperative paraplegia or weakness in two patients, and neurogenic bladder in one patient. Five patients (26.3%) developed minor complications after TES, including neuropathic pain in the T7 area in one patient, dural tear in one patient, wound dehiscence in one patient, and hardware failure in two patients. In the case of SABR, two patients (10.5%) experienced grade 3 toxicity after SABR, including weakness of the lower extremities in one patient suspected of having post-radiation myelitis and small bowel perforation resulting in laparotomy in one patient. Four patients (21.1%) developed grade 1 or 2 toxicity after SABR, including vertebral compression fracture (VCF) in two patients who were managed with conservative treatment, lower leg edema in one patient, and transient lower extremity weakness in one patient.

Using Cox regression analysis for all patients receiving SABR, we found that the significant risk factors associated with local progression after SABR included primary cancer of the liver or gallbladder (Hazard Ratio [HR] = 90.548, *p* < 0.001), having three or more lesions of extraspinal bone metastasis (HR = 81.440, *p* = 0.001), radioresistance of the primary cancer (HR = 65.106, *p* < 0.001), and Bilsky grade 3 metastasis (HR = 4.013, *p* = 0.025) (Table 3; full version in File S2). In the TES group, the analysis yielded no significant findings due to a limited number of patients.

## 4. Discussion

Our study found no discernible differences in the overall survival and local progression between patients undergoing TES and those receiving SABR for a single spinal metastasis. The TES group had a higher incidence of major complications than the SABR group. This inaugural study directly comparing exclusive SABR with TES demonstrated that SABR resulted in fewer complications and TES showed improved mid-term metastatic tumor control, yet there was no significant difference in overall survival between the groups.

In the matching process, five patients in the TES group did not have any exactly matching counterpart for tumor origin in the SABR group. Two poorly differentiated carcinomas and one adrenocortical carcinoma were classified as others or an unidentified class in the original article that employed a modified Tokuhashi score [25]. In this study, the other classes showed an average survival period of 6.7 months and the unidentified class showed an average survival period of 6.0 months. Therefore, these classes were matched with HCC, with an average survival period of 6.5 months. Likewise, thyroid cancer classified in the same score group as breast cancer could be matched with breast cancer in this study.

Our results showed no difference in the survival rates after 5 years of follow-up between the TES and SABR groups for single spinal metastasis (Figure 3). No previous study has directly compared the survival results of TES and SABR for single spinal metastasis [24]. Zheng et al. recently reported that SABR after separation surgery achieves outcomes comparable to TES for solitary, high-grade MESCC radioresistant metastatic spinal tumors, with shorter operation times and fewer perioperative complications [29]. This previous report aligns with our results, demonstrating that less invasive treatments, compared to TES, can achieve similar oncological outcomes with fewer adverse effects. However, our study was a direct comparison of the clinical outcomes of exclusive SABR without separation surgery and TES; therefore, further studies are warranted to generalize our results.

A recent systematic review of the oncological outcomes of TES in isolated spinal metastasis indicated that TES yields a low local recurrence rate of 6.1%, which is lower than the recurrence rate of 12.8% observed in our present study [23]. A meta-analysis of postoperative SABR data reported a one-year local recurrence rate of 11.1%, which is consistent with our findings [7]. In our analysis, the TES group demonstrated a superior initial local control compared to the SABR group, yet this distinction dissipated over time. The underreporting of initial local progression in the TES group can potentially be attributed to the interference caused by metal artifacts from the implanted materials, impacting image quality and obscuring the early detection of disease progression. The variation in local recurrence rates in our study can be attributed to the impact of tumor cell debulking. Pennington’s study indicated that extensive tumor debulking not only enhances mid-term but also long-term local control [30]. Specifically, they reported a 5-year local progression-free survival of 68.1% in patients undergoing anterior column debulking, compared to 0% in those with only epidural decompression. Their findings suggest that tumor cell debulking reduces local recurrence and bolsters long-term control. Our findings highlight the definitive role of surgical debulking in the midterm management of patients with single spinal metastasis. However, the impact on long-term survival outcomes appears to be unrelated to surgical debulking of spinal metastases. This discrepancy warrants further clarification through larger-scale studies.

In the TES group, four patients (21.1%) developed major complications after TES. A recent systematic review of the effect of TES for isolated spinal metastasis showed high complication rates (35.1%) owing to long surgical duration and significant blood loss that are higher than our results [23]. In the SABR group, only two patients (10.5%) experienced major complications of grade 3 toxicity after SABR. A recent meta-analysis of postoperative SABR in 461 patients reported one case of myelopathy and one case of esophageal fistula necessitating surgical repair [7]. Radiation myelopathy has been reported to have an incidence of less than 1% after SABR [31,32]. Our results showed an incidence of 5.3% for radiation myelopathy, which is much lower than the probability of nerve injury (15.8%) after TES, although the difference is not significant (*p* = 0.604). Previous studies on VCF after SABR showed that the rate of VCF was in the range of 11–39% with a crude VCF probability range of 2.4–13.9% [7,18]. In our study, only two patients had VCF. These results support the long-term safety of SABR over a follow-up period of 97.7 months.

Cox regression analysis of local tumor progression after SABR revealed that primary tumors with poor prognosis, three or more lesions with extraspinal bone metastasis, and radioresistance of the primary cancer were significant risk factors associated with local progression after SABR. These results are comparable with those of a previous study that advocated superior local tumor control in oligometastatic patients receiving SABR for spinal metastases compared to polymetastatic patients [17]. This may be explained by the nature of metastatic tumors, which represent a less aggressive oligometastatic phenotype. Given that HCC is a radioresistant tumor, its identification as a risk factor may be viewed through the lens of radioresistance. Previous studies have reported that histological type does not significantly correlate with local failure, and radioresistant histological types may benefit from SABR [16,33]. This discrepancy may be attributed to variations in total radiation dosage. In contrast to our study’s typical 18 Gy single-session SABR for HCC patients (Table 3), Yamada et al. utilized a higher 24 Gy dose and reported a 2% cumulative rate of local failure [16]. Consequently, our findings suggest that a single session of 24 Gy SABR is advisable for treating spinal metastasis in HCC patients.

Bilsky grade 3 metastasis was also a significant risk factor in our Cox regression analysis of local tumor progression. This result is echoed by previous studies showing that a higher grade of epidural disease, such as Bilsky grade 2 or 3, is associated with greater rates of local progression after postoperative SABR [34,35]. In the case of Bilsky grade 3 spinal metastases, the boundary between the spinal cord and the tumor is blurred, making it difficult to deliver a sufficient radiation dose to ablate the entire metastatic lesion while sparing the spinal cord with a dose limit of less than 16 Gy. In the context of Bilsky grade 3 cases for enhanced local tumor control, creating a gap between the spinal cord and the tumor appears to be necessary for the safe deployment of SABR.

Recent studies have examined the long-term outcomes of excisional surgeries, including TES and SBRT for single spinal metastasis. Clervide demonstrated that SBRT achieved 1, 2, and 3-year overall survival rates of 91.2%, 85.1%, and 83.2%, respectively [36]. Zheng found hybrid therapy’s survival and local progression-free survival rates comparable to TES at 1 year (84.6% vs. 83.1%), 2 years (60.8% vs. 64.3%), and 5 years (18.8% vs. 24.1%) [29]. Kato observed favorable outcomes with TES for spinal metastases from lung cancer, citing 3-, 5-, and 10-year survival rates of 61.5%, 53.8%, and 15.4%, respectively [37]. Cao noted 1-year survival rates of 84.6% for both TES and SABR following separation surgery, with no significant difference in overall survival between the groups [38]. Although direct comparison is difficult due to varying cancer types, a straightforward comparison of overall survival rates reveals comparable outcomes between patients treated with SABR and those undergoing surgical interventions, including TES and hybrid therapy. This observation, highlighting comparable survival outcomes between SABR and surgical modalities, aligns with our findings.

A critical point of this study was the potential distinction between indications for TES and SABR. Previous studies have questioned the efficacy of TES. However, recently, the distinction between the two treatments has become less clear. Traditionally, TES has been administered to patients with potentially curative treatment, whereas SABR was not initially used for curative intent in solitary spinal metastasis. Notwithstanding this assumed indication disparity that resulted in a potentially more curable patient group for TES, the lack of survival difference between the TES and SABR groups supports the significance of our findings. In response to the assumption that TES patients were treated earlier than those receiving SABR, our analysis confirms equivalent oncological statuses between the two groups. By matching tumor origin, NESMS, and modified Tokuhashi score in both TES and SABR groups, we established comparable oncologic conditions in our matched cohort. Our findings showed no significant variation in the duration from diagnosis of spinal metastasis to the commencement of treatment across these groups (Table 1). This indicates that TES and SABR were implemented at similar disease stages in our matched patient population.

Given the temporal disparity in treatment groups, the quality of adjunct chemotherapy might differ due to advancements in metastatic renal cell carcinoma management, transitioning from interleukin-2 and interferon-α to targeted immunotherapies [39]. In the TES group, two patients received conventional chemotherapy rather than the newer targeted therapies (File S3). This could potentially skew the survival rates in the TES and SABR cohorts. However, given that these patients also received active interventions, including en bloc resection, for any recurrent lesions, this variance in treatment is not expected to have significantly altered this study’s conclusions.

This study has several limitations. First, this was a retrospective study, and there is the possibility of confounding bias. Second, the number of matched pairs was too small to generalize the results. Therefore, a multicenter study with a larger sample size is required to generalize these results. Given the recent decline in TES surgeries, a multicenter study is anticipated to be a viable approach for accruing a sufficient patient cohort. Third, this study included primary tumors of heterogeneous origins. Each origin of the primary tumor has its own characteristics, and treatment should ideally be discussed for each cancer and its associated metastasis. A comparison between TES and SABR for a single spinal metastasis would be more accurate when limited to the same tumor origin.

## 5. Conclusions

Two-year local progression-free survival and overall survival rates were 66.7% and 78.9% in the TES group and 38.9% and 50.7% in the SABR group, respectively. The rate of major complications was higher in the TES group than in the SABR group (21.1% vs. 10.5%). At the final follow-up of the matched cohorts, no significant differences were noted in overall survival, progression-free survival, or local progression. TES and SABR both effectively managed solitary spinal metastasis. The TES group showed better midterm local tumor control than the SABR group, but long-term controls were similar. In conclusion, SABR resulted in fewer complications compared to TES, whereas TES demonstrated superior mid-term metastatic tumor control. Nonetheless, there was no significant difference in both overall survival and progression-free survival between the two treatment groups.

## Figures and Tables

**Table 1:** Demographic and oncologic characteristics of included patients and tumors.

Baseline Characteristics	All Patients			Matched Patients		
	TES (*n* = 20)	SABR (*n* = 69)	*p*-Value	TES (*n* = 19)	SABR (*n* = 19)	*p*-Value
Age (mean ± SD, years)	53.8 ± 13.3	57.4 ± 11.0	0.226	53.8 ± 13.6	61.8 ± 10.3	0.048
Sex, Male (%)	85.0	49.3	0.004	84.2	73.7	0.426
BMI (mean ± SD, kg/m^2^)	22.5 ± 2.2	22.5 ± 3.3	0.975	22.7 ± 2.2	22.1 ± 3.6	0.605
Follow-up period (mean ± SD, month)	56.6 ± 38.4	30.4 ± 25.8	0.008	55.7 ± 34.1	35.8 ± 28.5	0.050
Time from diagnosis of spinal metastasis to treatment (mean ± SD, month)	3.3 ± 6.8	3.0 ± 5.5	0.838	3.2 ± 7.0	2.7 ± 2.7	0.754
Involved Spinal level			0.215			0.615
Cervical	1 (5.0%)	9 (13.0%)		1 (5.3%)	2 (10.5%)	
Thoracic	13 (65.0%)	30 (43.5%)		12 (63.2%)	9 (47.4%)	
Lumbar	6 (30.0%)	30 (43.5%)		6 (31.6%)	8 (42.1%)	
Histology			0.003			0.412
RCC	6 (30.0%)	19 (27.5%)		6 (31.6%)	6 (31.6%)	
Thyroid	4 (20.0%)	2 (2.9%)		4 (21.1%)	2 (10.5%)	
Liver	3 (15.0%)	18 (26.1%)		3 (15.8%)	6 (31.6%)	
Breast	2 (10.0%)	21 (30.4%)		2 (10.5%)	4 (21.1%)	
NSCLC	1 (5.0%)	8 (11.6%)		1 (5.3%)	1 (5.3%)	
Esophageal	1 (5.0%)	0 (0.0%)		0 (0.0%)	0 (0.0%)	
Others	3 (15.0%)	1 (1.4%)		3 (15.0%)	0 (0.0%)	
SINS (median [IQR])	9.0 [5.25–10.75]	6.0 [5.0–9.0]	0.156	9.0 [5.0–10.0]	6.0 [6.0–10.0]	0.779
Tomita’s surgical classification			0.434			0.734
1	7 (35.0%)	23 (33.3%)		6 (31.6%)	6 (31.6%)	
2	8 (40.0%)	19 (27.5%)		8 (42.1%)	6 (31.6%)	
3	0 (0.0%)	0 (0.0%)		0 (0.0%)	0 (0.0%)	
4	5 (25.0%)	27 (39.1%)		5 (26.3%)	7 (36.8%)	
Metastases to internal organ			0.109			0.606
Nonremovable	5 (25.0%)	34 (49.3%)		5 (26.3%)	8 (42.1%)	
Removable	2 (10.0%)	8 (11.6%)		2 (10.5%)	1 (5.3%)	
None	13 (65.0%)	27 (39.1%)		12 (63.2%)	10 (52.6%)	
Number of extraspinal bone metastases foci			0.862			0.660
3 or more	0 (0.0%)	2 (2.9%)		0 (0.0%)	0 (0.0%)	
1–2	4 (20.0%)	17 (24.6%)		4 (21.1%)	2 (10.5%)	
0	16 (80.0%)	50 (72.5%)		15 (78.9%)	17 (89.5%)	
NESMS group						
1	0 (0.0%)	4 (5.8%)	0.202	0 (0.0%)	0 (0.0%)	1.000
2	5 (25.0%)	30 (43.5%)		5 (26.3%)	5 (26.3%)	
3	2 (10.0%)	3 (4.3%)		2 (10.5%)	2 (10.5%)	
4	13 (65.0%)	32 (46.4%)		12 (63.2%)	12 (63.2%)	
Modified Tokuhashi group						
1	2 (10.0%)	13 (18.8%)	0.627	2 (10.5%)	2 (10.5%)	1.000
2	11 (55.0%)	33 (47.8%)		10 (52.6%)	10 (52.6%)	
3	7 (35.0%)	23 (33.3%)		7 (36.8%)	7 (36.8%)	
ASA score						
1	5 (25.0%)	37 (53.6%)	0.017	5 (26.3%)	10 (52.6%)	0.184
2	14 (70.0%)	32 (46.4%)		13 (68.4%)	9 (47.4%)	
3	1 (5.0%)	0 (0.0%)		1 (5.3%)	0 (0.0%)	
ECOG scale						
0	9 (45.0%)	40 (58.0%)	0.067	9 (47.4%)	10 (52.6%)	0.070
1	11 (55.0%)	21 (30.4%)		10 (52.6%)	5 (26.3%)	
2	0 (0.0%)	8 (11.6%)		0 (0.0%)	4 (21.1%)	

TES, total en-bloc spondylectomy; SABR, stereotactic body radiation therapy; RCC, renal cell car-cinoma; NSCLC, non-small cell lung cancer; SINS, spinal instability neoplastic score; IQR, inter-quartile range; NESMS, New England Spinal Metastasis Score; ASA, American Society of Anaesthe-siologists; ECOG, Eastern Cooperative Oncology Group.

**Table 2:** The radiation regimens for the SABR group and the postoperative radiotherapy in the TES group.

Treatment Group	Time to Radiotherapy from TES (Week, Mean + SD)	Radiation Scheme	
		All Patients	Matched Patients
TES	4.1 ± 2.1	27 Gy in 3 fractions: 1 case 36 Gy in 6 fractions: 1 case 24 Gy in 6 fractions: 1 case 40 Gy in 16 fractions: 1 case 39 Gy in 13 fractions: 1 case 30 Gy in 10 fractions: 5 cases 44 Gy in 22 fractions: 1 case	27 Gy in 3 fractions: 1 case 36 Gy in 6 fractions: 1 case 24 Gy in 6 fractions: 1 case 40 Gy in 16 fractions: 1 case 39 Gy in 13 fractions: 1 case 30 Gy in 10 fractions: 5 cases 44 Gy in 22 fractions: 1 case
Treatment group	Primary cancer type	All patients	Matched patients
SABR	RCC	24 Gy in 1 fraction: 1 case 18 Gy in 1 fraction: 16 cases 16 Gy in 1 fraction: 2 cases	18 Gy in 1 fraction: 5 cases 16 Gy in 1 fraction: 1 cases
	Thyroid	18 Gy in 1 fraction: 2 cases	18 Gy in 1 fraction: 2 cases
	HCC	20 Gy in 1 fraction: 1 case 18 Gy in 1 fraction: 6 cases 30 Gy in 3 fractions: 1 case 27 Gy in 3 fractions: 4 cases 24 Gy in 3 fractions: 5 cases 24 Gy in 4 fractions: 1 case	18 Gy in 1 fraction: 1 case 24 Gy in 3 fractions: 4 cases
	Breast	20 Gy in 1 fraction: 1 case 18 Gy in 1 fraction: 19 cases 24 Gy in 3 fractions: 1 cases	20 Gy in 1 fraction: 1 case 18 Gy in 1 fraction: 2 cases 24 Gy in 3 fractions: 1 cases
	NSCLC	20 Gy in 1 fraction: 1 case 18 Gy in 1 fraction: 3 cases 30 Gy in 3 fractions: 2 case 27 Gy in 3 fractions: 1 cases 35 Gy in 5 fractions: 1 cases	30 Gy in 3 fractions: 2 case
	Others	18 Gy in 1 fraction: 1 cases	

TES, total en-bloc spondylectomy; SD, standard deviation; SABR, stereotactic body radiation ther-apy; RCC, renal cell carcinoma; HCC, hepatocellular carcinoma; NSCLC, non-small cell lung can-cer; Gy, gray.

**Table 3:** Cox regression analyses about local progression for all patients treated with SABR.

	Univariate Analysis		Multivariate Analysis	
	Hazard Ratio (95% CI)	*p* Value	Hazard Ratio (95% CI)	*p* Value
Sex (male)	2.238 (0.971–5.157)	0.059		0.096
Primary cancer group of modified Tokuhashi score		0.001		<0.001
Kidney, uterus ^a^	3.381 (0.907–12.611)	0.070		0.051
Liver, gallbladder ^a^	12.994 (3.459–48.813)	<0.001	90.548 (10.146–808.107)	<0.001
Lung, pancreas, etc. ^a^	3.780 (1.078–13.251)	0.038		0.051
Extraspinal bone metastasis		0.045		0.048
1–2 ^b^		0.331		0.968
≥3 ^b^	15.505 (1.668–144.133)	0.016	81.440 (5.402–1227.676)	0.001
Radioresistance of the primary cancer	1.915 (0.824–4.450)	0.131	65.106 (7.424–570.990)	<0.001
Prior radiotherapy	4.381 (1.253–15.324)	0.021		0.064
High bilsky grade	2.862 (1.207–6.788)	0.017		0.305
Bilsky grade 3	2.991 (1.148–7.793)	0.025	4.013 (1.191–13.473)	0.025

^a^ Hazard ratio compared to primary cancer origin of thyroid, breast, prostate carcinoid tumor. ^b^ Hazard ratio compared to no extraspinal bone metastasis.

## Data Availability

Data underlying this article cannot be shared publicly due to the privacy of the individuals that participated in this study. The data may be shared on reasonable request to the corresponding author.

## Supplementary Materials

The following supporting information can be downloaded at: https://www.mdpi.com/article/10.3390/cancers15235518/s1, File S1: Oncological data of the matched patients; File S2: Cox regression analyses about local progression for all patients treated with SABR. File S3: Adjuvant chemotherapy of the matched patients.
