# Supplementary material for: Efficacy of Total En Bloc Spondylectomy versus Stereotactic Ablative Radiotherapy for Single Spinal Metastasis
Source: Cancers (Basel). 2023 Nov 22;15(23):5518. doi: 10.3390/cancers15235518 (PMC10705804; doi:10.3390/cancers15235518)
Supplement: Supplementary file 1 [file cancers-15-05518-s001.zip › File S1.pdf]

File S1. Oncological data of the matched patients

Pair no./sex/ age (year)/ Tx.	Entities	Affected vertebra	Primary tumor site	New England spinal metastasis score	Modified Tokuhashi score	Other metastases site at the time of Tx.	Follow -up (mo)	Local recurrence	Time to local recurrence (mo)	Overall survival from Tx. (mo)	Status
1 / F / 27 / TES	RCC	L4	Right kidney	4	13	None	54.4	No		55.2	DOD
1 / M / 84 / SABR	RCC	C3	Right kidney	4	12	None	26.1	No		44.3	AWD
2 / M / 77 / TES	RCC	T11	Left kidney	4	11	Clavicle	56.2	No		57.6	DOD
2 / M / 80 / SABR	RCC	T2	Left kidney	4	10	Lung, mediastinal Lymph node	21.7	No		22.8	DOD
3 / M / 61 / TES	Thyroid cancer	L1		4	14	Lung	97.7	No		97.7	NED
3 / F / 62 /7A SABR	Thyroid cancer	T12		4	15	None	69.0	Yes	17.0	87.0	AWD
4 / M / 64 / TES	Poorly differentiated carcinoma	T3	T3	4	9	None	26.3	No		27.4	DOD
4 / M /66 / SABR	HCC	L1		4	9	None	3.6	No		6.7	DOD
5 / M / 56 / TES	HCC	T7		2	7	Lung, ilium, rib	9.6	No		11.4	DOD
5 / F / 67 / SABR	HCC	L3		2	7	Lung, ilium	39.8	No		41.0	DOD
6 / M / 71 / TES	Thyroid cancer	T9		4	15	None	95.4	No		96.6	DOD
6 / F / 58 / SABR	Breast cancer	T11	Left breast	4	15	None	89	No		94.5	AWD
7 / M / 69 / TES	HCC	L1		3	9	None	18.7	No		19.4	DOD
7 / M / 55 / SABR	HCC	C1		3	11	None	21.7	Yes	8.9	22.2	DOD

8 / F / 48 / TES	Breast cancer	L2	Left breast	4	15	None	91.6	No		93.3	DOD
8 / F / 50 / SABR	Breast cancer	T2	Right breast	4	15	None	42.3	No		47.2	NED
9 / M / 61 / TES	RCC	L2	Left kidney	3	10	Lung	20.2	No		23.7	DOD
9 / M / 64 / SABR	RCC	L2	Left kidney	3	10	Lung	9.6	Yes	9.2	10.8	DOD
10 / M / 66 / TES	RCC	T9	Left kidney	4	11	Pancreas	31	No		31.8	DOD
10 / M / 69 / SABR	RCC	L4	Right kidney	4	10	Lung	17.8	No		18.5	DOD
11 / M / 52 / TES	HCC	T12		4	11	None	25.8	No		21.4	DOD
11 / M / 56 / SABR	HCC	T9		4	11	None	13.0	Yes	11.9	13.6	DOD
12 / F / 54 / TES	Breast cancer	T4	Left breast	4	14	None	97.7	No		97.7	NED
12 / F / 53 / SABR	Breast cancer	T7	Left breast	4	14	LN	51.4	No		55.8	NED
13 / M / 56 / TES	Thyroid cancer	T8		2	13	Scapula, rib	73.7	No		86.8	NED
13 / M / 62 / SABR	Thyroid cancer	T4		2	12	Lung	14.5	Yes	9.9	16.2	DOD
14 / M / 38 / TES	RCC	T6	Left kidney	2	10	Femur, adrenal, Lymph node	64.7	No		66.7	DOD
14 / M / 78 / SABR	RCC	L2	Right kidney	2	10	Lung, scapula, rib	27.1	No		36.8	AWD
15 / M / 32 / TES	Adrenal cortical carcinoma	T11		4	11	None	46.6	No		47.5	DOD
15 / M / 59/ SABR	HCC	T5		4	11	None	16.5	Yes	1.4	16.5	DOD
16 / M / 51 / TES	RCC	T5	Right kidney	4	11	None	97.7	Yes	44.6	97.7	AWD

16 / M / 55 / SABR	RCC	T5	Left kidney	4	11	Lung	34	Yes	12.6	40.1	AWD
17 / M / 53 / TES	Thyroid cancer	T1		4	15	None	97.7	Yes	61.3	97.7	AWD
17 / F / 50 / SABR	Breast cancer	L4	Left breast	4	14	None	59.7	No		64.2	AWD
18 / M / 33 / TES	Poorly differentiated carcinoma	L4	L4	2	7	Lung	26.4	Yes	20.5	47.6	DOD
18 / M / 47 / SABR	HCC	L3		2	8	Lung	0.9	No		2.5	DOD
19 / M / 53 / TES	NSCLC	C7		2	10	None	13.5	Yes	6.2	13.8	DOD
19 / M / 60 / SABR	NSCLC	L3		2	10	None	79	No		80.4	DOD
