# Supplementary material for: Efficacy of Total En Bloc Spondylectomy versus Stereotactic Ablative Radiotherapy for Single Spinal Metastasis
Source: Cancers (Basel). 2023 Nov 22;15(23):5518. doi: 10.3390/cancers15235518 (PMC10705804; doi:10.3390/cancers15235518)
Supplement: Supplementary file 1 [file cancers-15-05518-s001.zip › File S2.pdf]

File S2. Cox regression analyses about local progression for all patients treated with SABR.

	Univariate analysis		Multivariate analysis	
	Hazard ratio (95% CI)	*P* value	Hazard ratio (95% CI)	*P* value
Age (years)		0.352		
Sex (male)	2.238 (0.971-5.157)	0.059		0.096
ASA		0.213		
ECOG≥2	2.768 (0.793-9.665)	0.110		
Primary cancer group of modified Tokuhashi score		0.001		**<0.001**
Kidney, uterus [a]	3.381 (0.907-12.611)	0.070		0.051
Liver, gallbladder [a]	12.994 (3.459-48.813)	<0.001	**90.548 (10.146-808.107)**	**<0.001**
Lung, pancreas,etc [a]	3.780 (1.078-13.251)	0.038		0.051
Extraspinal bone metastasis		0.045		**0.048**
1-2 [b]		0.331		0.968
≥3 [b]	15.505 (1.668-144.133)	0.016	**81.440 (5.402-1227.676)**	**0.001**
Internal organ metastasis		0.013		0.268
Removable [c]		0.352		0.431
Unremovable [c]	2.801 (1.131-6.936)	0.026		0.113
Radioresistancy	1.915 (0.824-4.450)	0.131	**65.106 (7.424-570.990)**	**<0.001**
Location of spinal metastasis		0.609		
Thoracic [d]		0.323		
Cervical [d]		0.780		
Prior radiotherapy	4.381 (1.253-15.324)	0.021		0.064
Prior adjuvant Tx.		0.865		
Total radiation dose		0.227		
Fraction		0.204		
High bilsky grade	2.862 (1.207-6.788)	0.017		0.305
Bilsky grade 3	2.991 (1.148-7.793)	0.025	**4.013 (1.191-13.473)**	**0.025**
Size > 30mm		0.423		
Time from diagnosis to treatment (month)		0.229		

[a] Hazard ratio compared to primary cancer origin of thyroid, breast, prostate carcinoid tumour, [b] Hazard ratio compared to no extraspinal bone metastasis, [c] Hazard ratio compared to no internal organ metastasis, [d] Hazard ratio compared to lumbar.
