# Supplementary material for: Efficacy of Total En Bloc Spondylectomy versus Stereotactic Ablative Radiotherapy for Single Spinal Metastasis
Source: Cancers (Basel). 2023 Nov 22;15(23):5518. doi: 10.3390/cancers15235518 (PMC10705804; doi:10.3390/cancers15235518)
Supplement: Supplementary file 1 [file cancers-15-05518-s001.zip › File S3.pdf]

File S3. Adjuvant chemotherapy of the matched patients

Pair no./sex/ age (year)/ Tx.	Primary cancer type	Adjuvant chemotherapy agent and cycles (#N)	Period
1 / F / 27 / TES	RCC	VAC/IE #5<br>Cytoxan/Topotecan #4	-2001.11<br>2004.7-2004.10
1 / M / 84 / SABR	RCC	Pazopanib	2019.2-2021.3
2 / M / 77 / TES	RCC	None	
2 / M / 80 / SABR	RCC	None	
3 / M / 61 / TES	Thyroid cancer	RAI #12	-2010.8
3 / F / 62 /7A SABR	Thyroid cancer	RAI #7	2007.6-2016.8
4 / M / 64 / TES	Poorly differentiated carcinoma	None	
4 / M /66 / SABR	HCC	Sorafenib<br>Regorafenib<br>Nivolumab	2018.12-2019.1<br>2019.1-2019.4<br>2019.5-2019.7
5 / M / 56 / TES	HCC	Sorafenib	2012.1-2012.3
5 / F / 67 / SABR	HCC	Sorafenib<br>Regorafenib<br>PD1 + LAG3 #10<br>5-fluorouracil/Cis-platinum #5<br>Adriamycin #1	2017.7-2018.7<br>2018.10-2019.5<br>2019.9-2020.4<br>2020.4-2020.9<br>2020.9-11
6 / M / 71 / TES	Thyroid cancer	RAI #5	-2013.11
6 / F / 58 / SABR	Breast cancer	Zoladex/Tamoxifen<br>Latrozole<br>Aromasin #24<br>Faslodex #21<br>Capecitabine #39<br>Paclitaxel #6<br>Gemcitabine/Cisplatin #13	2012.7<br>2012.7-2014.12<br>2014.12-2017.4<br>2017.5-2017.10<br>2019.1-2021.7<br>2021.8-2022.3<br>2022.3-
7 / M / 69 / TES	HCC	Nexavar #6	-2012.6
7 / M / 55 / SABR	HCC	Sorafenib<br>Nivolumab	2019.5-2019.12<br>2020.2-2021.2
8 / F / 48 / TES	Breast cancer	Tamoxifen #10<br>Docetaxel #28	-2016.3<br>-2018.9
8 / F / 50 / SABR	Breast cancer	Tamoxifen<br>Latrozole<br>Xeloda #69	2014.9-2018.4<br>2018.7-2018.12<br>2018.12-
9 / M / 61 / TES	RCC	Sunitinib #5<br>Everolimus #11	2013.2-2013.9<br>2013.10-2014.8
9 / M / 64 / SABR	RCC	Bevacizumab<br>Sunitinib<br>Everolimus	2015.8-2017.3<br>2017.4-2017.5<br>2017.5-2017.6
10 / M / 66 /	RCC	Sunitinib #20	2011.1-2013.3

TES		RAD001 #3<br>Sorafenib #3	2013.6-2013.8<br>2013.9-2013.12
10 / M / 69 / SABR	RCC	Pazopanib<br>Axitinib<br>Cabozantinib<br>Everolimus	2017.1-2019.2<br>2019.2-2019.12<br>2019.12-2020.5<br>2020.5-2021.6
11 / M / 52 / TES	HCC	FOLFOX #2<br>Paclitaxel/Cisplatin #8<br>FOLFIRI #6	2013.11-2013.12<br>2014.1-2014.6<br>2014.7-2014.10
11 / M / 56 / SABR	HCC	Sorafenib<br>Regorafenib<br>Nivolumab	2019.1-2019.3<br>2019.3-2019.6<br>2019.6-2019.9
12 / F / 54 / TES	Breast cancer	Adriamycin/Cyclophosphamide #4<br>Paclitaxel #4<br>Xeloda #8<br>Verzenio/Faslodex #25-27	 -2014.4<br>-2019.6<br>2020.7-
12 / F / 53 / SABR	Breast cancer	Docetaxel #8	2017.6-2017.11
13 / M / 56 / TES	Thyroid cancer	RAI #1	2016.1
13 / M / 62 / SABR	Thyroid cancer	RAI #1<br>Sorafenib<br>Lenvatinib<br>Adriamycin/Cisplatin	2015.1<br>2015.2-2016.3<br>2016.7-2019.10<br>2019.12-2020.2
14 / M / 38 / TES	RCC	Sunitinib #16<br>Atezolizumab/Cobimetinib<br>Axitinib<br>Cabozantinib	-2017.12<br>2017.12-2018.7<br>2018.7-2019.4<br>2019.5-2019.7
14 / M / 78 / SABR	RCC	Pazopanib	2019.3-2021.12
15 / M / 32 / TES	Adrenal cortical carcinoma	Mitotane<br>Doxorubicin/Cisplatin #6	2014.1-2014.7<br>2014.10-2015.1
15 / M / 59/ SABR	HCC	Regorafenib<br>Cabozatinib	2018.11-2019.1<br>2019.12-2020.3
16 / M / 51 / TES	RCC	Sunitinib<br>Everolimus<br>Cabozantinib<br>Nivolumab #2	2014.3-2017.3<br>2017.3-2019.5<br>2021.12-2022.1<br>2022.1-2022.2
16 / M / 55 / SABR	RCC	Pazopanib<br>Sunitinib<br>Axitinib<br>Cabozantinib	2018.12-2018.1<br>2019.2-2020.11<br>2020.12-2021.9<br>2021.9-
17 / M / 53 / TES	Thyroid cancer	RAI #2	2020.4-2021.1
17 / F / 50 / SABR	Breast cancer	TCHP/Neulapeg #6<br>Herceptin #8<br>TDM-1 #15<br>Eribulin/Trastuzumab #24<br>Trastuzumab Deruxtecan #42	2016.6-9<br>2016.11-2017.4<br>2017.7-2018.7<br>2018.9-2020.1<br>2020.3-
18 / M / 33 / TES	Poorly differentiated carcinoma	Docetaxel/Cyclphosphamide #4	2015.8-2015.10
18 / M / 47 / SABR	HCC	Sorafenib	2017.5-2018-9

19 / M / 53 / TES	NSCLC	Cisplatin/Vinorelbine #4<br>Gefitinib<br>Cisplatin/Gemcitabine #2<br>Paclitaxel/Carboplatin #2<br>Pemetrexed #2	2011.11-2012.1<br>2012.9-2013.3<br>2013.5-2013.6<br>2013.6-2013.8<br>2013.8-2013.9
19 / M / 60 / SABR	NSCLC	LXH254#2	2019.1-2
